# Neutral Lipid Storage Diseases: clinical/genetic features and natural history in a large cohort of Italian patients

**DOI:** 10.1186/s13023-017-0646-9

**Published:** 2017-05-12

**Authors:** Elena Maria Pennisi, Marcello Arca, Enrico Bertini, Claudio Bruno, Denise Cassandrini, Adele D’amico, Matteo Garibaldi, Francesca Gragnani, Lorenzo Maggi, Roberto Massa, Sara Missaglia, Lucia Morandi, Olimpia Musumeci, Elena Pegoraro, Emanuele Rastelli, Filippo Maria Santorelli, Elisabetta Tasca, Daniela Tavian, Antonio Toscano, Corrado Angelini, The Italian NLSD Group

**Affiliations:** 1 UOC of Neurology, San Filippo Neri Hospital, via Martinotti 20, 00135 Rome, Italy; 2 Department of Internal Medicine and Allied Sciences, Atherosclerosis Unit, Sapienza University of Rome, Rome, Italy; 3 IRCCS Bambin Gesù Hospital, Rome, Italy; 4 IRCCS Gaslini, Genova, Italy; 5 IRCCS Fondazione Stella Maris, Calambrone, Pisa, Italy; 6 S. Andrea Hospital, La Sapienza University of Rome, Rome, Italy; 7 Sandro Pertini Hospital, Neurology, Rome, Italy; 8 Neuroimmunology and Neuromuscular Diseases Unit, Foundation IRCCS Neurological Institute “Carlo Besta”, Milan, Italy; 9 Department of Systems Medicine, Centre of Neuromuscular Disorders, Tor Vergata University, Rome, Italy; 10 CRIBENS, Catholic University of the Sacred Heart, Milan, Italy; 11 Department of Neurosciences, University of Messina, Messina, Italy; 12 Department of Neurology, University of Padova, Padova, Italy; 13 IRCCS Fondazione Ospedale S. Camillo, Venice, Italy

**Keywords:** NLSD, PNPLA2, CGI58, Myopathy, Lipid metabolism, Natural history

## Abstract

**Background:**

A small number of patients affected by Neutral Lipid Storage Diseases (NLSDs: NLSD type M with Myopathy and NLSD type I with Ichthyosis) have been described in various ethnic groups worldwide. However, relatively little is known about the progression and phenotypic variability of the disease in large specific populations. The aim of our study was to assess the natural history, disability and genotype-phenotype correlations in Italian patients with NLSDs. Twenty-one patients who satisfied the criteria for NLSDs were enrolled in a retrospective cross-sectional study to evaluate the genetic aspects, clinical signs at onset, disability progression and comorbidities associated with this group of diseases.

**Results:**

During the clinical follow-up (range: 2–44 years, median: 17.8 years), two patients (9.5%, both with NLSD-I) died of hepatic failure, and a further five (24%) lost their ability to walk or needed help when walking after a mean period of 30.6 years of disease. None of the patients required mechanical ventilation. No patient required a heart transplant, one patient with NLSD-M was implanted with a cardioverter defibrillator for severe arrhythmias.

**Conclusion:**

The genotype/phenotype correlation analysis in our population showed that the same gene mutations were associated with a varying clinical onset and course. This study highlights peculiar aspects of Italian NLSD patients that differ from those observed in Japanese patients, who were found to be affected by a marked hypertrophic cardiopathy. Owing to the varying phenotypic expression of the same mutations, it is conceivable that some additional genetic or epigenetic factors affect the symptoms and progression in this group of diseases.

## Background

The triglycerides (TG) are involved in the synthesis and degradation pathways of lipids, they are essential for energy production and for the synthesis of important cellular structures [1]. The TG not only contribute to energy production in adipose tissue during fasting, but also in skeletal muscle during physical exercise. Some enzymes allow the release of triglycerides from the lipid droplets in the cytoplasm, two of the most important are adipose triglyceride lipase (ATGL/PNPLA2, MIM 609059) and comparative gene identification-58 (CGI-58/ABHD5, MIM 604780) [2, 3]. Inborn errors affecting ATGL and CGI58 cause two different diseases: Neutral Lipid Storage Disease with Myopathy (NLSD-M, MIM 610717) and Neutral Lipid Storage Disease with Ichthyosis (NLSD-I, Chanarin-Dorfman disease, MIM 604780). Neutral Lipid Storage Diseases (NLSDs) are rare autosomal recessive disorders characterized by excessive, non-lysosomal, accumulation of neutral lipids in multiple tissues. Clinically NLSDs cause muscle atrophy, cardiomyopathy, dysfunction of several internal organs as well as ichthyosis. The animal model of disease is more severe than the human. Structural defects in the PNPLA2 gene mainly lead to myopathic symptoms, whereas mutations in the CGI58 gene, the activator of PNPLA2, mainly cause ichthyosis and hepatic symptoms associated with myopathic symptoms. Although the biochemical basis and pathogenesis of NLSDs are only partially understood, it is known that these two enzymes release TG from cytoplasmic lipid droplets to supply beta-oxidation in energy production into mitochondria and to assemble cellular membranes. Lipid accumulation is present in the skin, muscle, liver, thyroid, pancreas, heart, central nervous system and leukocytes. NLSDs are characterized, among other things, by lipid-containing vacuoles in white blood cells (named “Jordans’ anomaly”, from first observer in 1953) [4], which are considered the main diagnostic hallmark of NLSDs. Along with important clinical features, like hepatic steatosis, skeletal myopathy and cardiomyopathy, less frequently are present bilateral cataracts, growth retardation, ataxia, bilateral sensorineural hearing loss and intellectual disability. Sporadic and familial forms of NLSDs with a pan-ethnic distribution have been described, though detailed descriptions of large numbers of patients and proper genotype-phenotype relationships are lacking. The frequency of mutations and the mechanisms leading to muscle damage also remain largely unknown. The geographic dispersion of the very small number of patients and the difficulty of diagnosis further hamper research in this field. The aim of this study is to create an Italian registry of NLSD patients to determine their phenotypes and natural history as well as to investigate any genetic-phenotypic correlations by collecting clinical and molecular findings.

## Methods

Fourteen centers agreed to take part in this retrospective study, which started in 2013, during the annual meeting of the Italian Myology Association. Nine neuromuscular centres (geographically covering whole Italy) selected patients of all ages with lipid myopathy from their own databases. Similar disorders characterized by excessive lipid storage, i.e. riboflavin-responsive MAD deficiency due to ETF-dehydrogenase mutations, carnitine disorders and mitochondrial disorders, were excluded. NLSDs in all the patients were confirmed by means of genetic tests.

All diagnostic procedures followed the standard principles and were approved by the local ethics committees of all the participating neuromuscular centers in agreement with the Helsinki Declaration of 1975, revised in 2000. The patients gave their informed consent to the genetic investigation and to the publication of photos. The following clinical data were analyzed: age, sex, onset of initial symptoms as reported by patients, signs and symptoms of muscle weakness and atrophy, daily living activities, respiratory function evaluated by means of spirometry and, when possible, the six-minute walking test, skin, endocrine and cardiac involvement, laboratory data such as blood tests (CK, serum lipids, glycaemia), EMG, ECG and Holter ECG, echocardiography, internal organ ultrasound, respiratory performance and cause of death. The inclusion criteria were: 1) lipid storage myopathy in patients or family members, 2) Jordans’ anomaly, 3) presence of mutations in the PNAPLA2 or CGI58 genes. Genetic analyses were performed in three Italian centers (Milan, Rome and Pisa). Genomic DNA was extracted from peripheral blood using a Puregene DNA Isolation kit (Gentra Systems, Minneapolis). The coding region of the PNPLA2 gene (GeneBank NM02376) was amplified using the oligonucleotides and PCR amplification conditions previously reported by Tavian et al. [5]. All CGI58/ABHD5 coding exons (GeneBank NG007090.3) and the candidate promoter region were PCR amplified. The conditions for the genomic amplification followed those described by Redaelli et al. [6]. All PCR products were gel purified (NucleoSpin Extract II, M-Medical) and sequenced on 3730 DNA Analyzers by means of the BigDye® Terminator V1.1 Cycle Sequencing Kit (Applied Biosystems, Foster City, CA).

To confirm the diagnosis, all the patients were tested for Jordans’ anomaly using peripheral blood collected by means of finger or brachial vein puncture; the smeared slides were then stained using the Giemsa method to verify the presence of lipid vacuoles in leucocytes by immersion optic microscopy (100X). Muscle biopsies were available in 11/15 patients with NLSD-M and 3/6 patients with NLSD-I. The muscle biopsy was not carried out in four NLSD-M patients whose relatives had already been biopsied and in three patients with NLSD-I, two of whom mainly displayed hepatic symptoms. Morphological studies were performed on muscle tissue obtained by open biopsy: cryosections were stained following standard histochemical and immunohistochemical procedures.

Patients were examined by means of instrumental and clinical tests throughout the follow-up period in their referral neuromuscular centers by neurologists and cardiologists. All the patients underwent annual clinical and neurological examination. Muscle strength of the upper limbs, lower limbs and axial muscles was tested by means of the Medical Research Council (MRC) scale.

Myalgia, fatigue, swallowing and dysphagia were assessed by asking patients specific questions on these disorders at each follow-up visit. At least one EMG/ENG study was performed in 14 patients with NLSD-M, and in 2 patients with NLSD-I.

## Results

Clinical, genetic and instrumental data were collected from 21 patients: 15 patients with NLSD-M (9 men and 6 women, age range: 14–80 year, Table 1) and 6 patients with NLSD-I (1 man and 5 women, age range: 16–69 years, Table 2). Patients were followed up for a mean period of 17.8 years (range: 2–44 years). The clinical diagnosis was made at an age ranging between 1 and 66 years Most patients originated from regions in the centre and south of Italy (Lazio, Sardinia, Molise, Puglia and Sicilia); one patient (pt.V.1) was born in Iran, but was diagnosed in Italy where she has been living stably for the past 30 years.

**Table 1:** Clinical findings of NLSD-M patients

References		CaM [Tavian, 2012] [5]	CaAB [Tavian, 2012] [5]	MA This report	DLA [Campagna, 2008] [7]	DLC [Campagna, 2008] [7]	DE [Pennisi, 2015] [8]	RR [Campagna, 2008] [7]	GA [Pasanisi, 2016] [10]	RMC This report	BL [Missaglia, 2015]	BP [Missaglia, 2015]	BMC [Missaglia, 2015] [9]	RC [Tavian, 2012] [5]	CM68 [Massa, 2016] [11]	AZ [Fiorillo, 2013] [12]
Family		I	I	II	III	III	IV	V	VI	VII	VIII	VIII	VIII	IX	X	XI
Patient		I.1	I.2	II.1	III.1	III.2	IV.1	V.1	VI.1	VII.1	VIII.1	VIII.2	VIII.3	IX.1	X.1	XI.1
Sex		F	M	F	M	M	F	F	M	F	M	M	F	M	M	M
Onset of symptoms		25y	40y	47y	34y	35y	58y	18y	1y	40y	40y	35y	58y	64y	45y	5y
First symptom		M	M	M	M	M	M	M	M	M	M	M	HF	M	M	M
Years of disease		44	22	24	16	10	16	34	26	13	20	15	2	15	2	9
Age at last clinical examination		69y	62y	74y	50y	45y	74y	52y	26y	53y	60y	50y	60y	79y	47y	14y
Weakness	proximal	UL*, LL	UL, LL	UL > LL	UL* LL	UL, LL	LL	UL*, LL	UL, LL	UL* > LL	UL	-	UL	UL	-	-
	distal	UL, LL	UL, LL	UL > LL	UL, LL	UL, LL	-	UL	LL, UL	-	-	UL, LL	-	UL	LL	-
	axial	+	-	-	+	+	-	+	-	-	+	-	-	+	-	-
Other clinical features		-	-	-	SW	-	C	-	TTW	SW, CH	RI	SW	-	DH, HL	-	-
Exercise intolerance		+	+	+	+	+	+	+	+	+	+	+	-	-	+	-
Myalgias		+	+	-	-	-	+	-	+	-	-	+	-	-	+	-
Muscle cramps		+	+	-	-	-	-	-	-	-	+	+	-	-	-	-
Spine deformities		-	-	-	-	S	S	-	-	-	K	S	-	-	-	-
Cardiac involvement		HCM	-	HCM	LVHT	HCM	-	-	HCM	-	-	-	-	-	HCM	-
PMK/ICD implantation		-	-	-	PMK	-	-	-	-	-	-	-	-	-	-	-
Hepatic steatosis		+	-	-	+	+	-	+	-	-	+	+	+	+	+	-
Ichthyosis		-	-	-	transient	-	-	-	-	-	-	-	-	-	-	-
Jordans’ anomaly		100%	90%	100%	100%	100%	100%	10%	100%	100%	100%	100%	75%	100%	100%	100%
Age at muscle biopsy		44y	n. p	57y	35y	n. p	58y and 72y	40y	8y and 21y	49y	39y and 40y	37y	n. p	70y and 71y	44 y	14y
Lipidosis in muscle biopsy		+	n. p	+	+	n. p.	-	+	+	+	+	+	n. p	+	+	+
EMG		My	Mp, My	My	Mp, My, SA	Mp, My	Mp, My	Mp, N	Mp	SA	Mp	n. p	n. p	N	Mp	Mp
Max CK value		4X	2.5X	8X	5X	3X	1.5X	5X	25X	3x	5X	6X	6X	2X	3X	6X

*M* muscular, *H* Hepatic, *UL* Upper Limbs, *LL* Lower Limbs, *asymmetrical weakness, -: not, +: yes, *L* Lordosis, *K* kyphosis, *S* scoliosis, *DCM* dilated cardiomyopathy, *HCM* hypertrophic cardiomyopathy, *LVHT* left ventricular hypertrabeculation (noncompaction), *HF* hepatic failure, *RI* respiratory involvement, *TTW* tip-toe walking, *SW* scapular winging, *DH* drop head, *CH* calf hypertrophy, *HL* hearing loss, *C* cataract, *PMK/ICD* pacemaker/implantable cardioverter; Jordans’ anomaly: percentage of leukocytes that showed the anomaly; n. p: not performed, *Mp*: myopathic, *My* myotonia, *SA* spontaneous activity, *N* neuropathic

**Table 2:** Clinical findings in NLSD-I patients

Patients		LG	VG	CM39	SF	AA	LB
References		[Bruno, 2008] [13]	[Bruno 2008] [13]	[Gaeta, 2008] [14]	[Redaelli,2010-Ronchetti, 2008] [6, 18]	[Redaelli, 2010] [6]	[Angelini, 1980] [16]
Family		XII.1	XII.2	XIII.1	XIV.1	XV.1	XVI.1
Mutation		p.R184X/p.R184X. loss of α/β hydrolase domain	p.R184X/p.R184X. loss of α/β hydrolase domain	IVS4-1G > A Probably not functional protein	c.47 + 1G > A Probably no protein production		p. S33X/p.R297X. Probably no protein production/ It loses the end of α/β hydrolase domain and C-terminal domain
Sex		M	F	F	F	M	F
Onset of symptoms		Birth	Birth	Birth	Birth	Birth	5y
First symptom		C	C	C	C	C	H
Age at last clinical examination		15y	28y	69y	42y	16y	5y
Weakness	proximal	-	-	LL	-	-	UL, LL
	distal	-	-	UL, LL	-	-	UL, LL
	axial	-	-	-	-	-	+
Other clinical features		-	-	HL	CT	-	-
Exercise intolerance		-	-	+	-	-	-
Myalgias		-	-	-	-	-	-
Muscle cramps		-	-	+	-	-	-
Spine deformities		-	-	L	-	-	L
Cardiac involvement		-	-	HCM	-	-	-
PMK/ICD implantation		-	-	-	-	-	-
Liver involvement		+	+	-	+	+	+
Ichthyosis		+	+	+	+	+	+
Jordans’ anomaly		n.p.	n.p.	+	+	+	+
Age at muscle biopsy		6 y	n.p.	65y	n.p.	n.p.	5y
Lipidosis in muscle biopsy		+	n.p.	+	n.p.	n.p.	+
EMG		n.p.	n.p.	N, SA	n.p.	n.p.	M
Max CK value		3.5X	1.5X	2X	Normal	Normal	1.5X

*C* Cutaneous, *H* Hepatic, *LL* Lower Limbs, *UL* Upper Limbs, +: yes, -: not, *HL* hearing loss, *CT* cataract, *L* Lordosis, *HCM* hypertrophic cardiomyopathy, *M* myopathic features, *SA* spontaneous activity, *N* neuropathic features, *n.p* not performed

Table 3 shows the results of the age/genotype/clinical severity correlation analysis in 4 groups of NLSD-M patients divided according to the degree of muscle involvement: severe (loss of ambulation, use of wheelchair); moderate (interference with daily activities, e.g. weakness when climbing stairs); mild (muscle weakness but no interference with daily activities); asymptomatic (hyperCKemia without symptoms).

**Table 3:** Clinical-genetic correlation in NLSD-M patients

Family	Patient	Age at onset	Sex and age	DNA mutations in PNPLA2 gene	Protein mutation	Mutation effect	Clinical severity
Family I	I.1	25y	F, 69y	c.24G > C	PT	Probably no protein production	Severe
				c.516C > A	MM	Conserve localization and partially lipase function	
	I.2	40y	M, 62y	c.24G > C	PT		Moderate
				c.516C > A	MM		
Family II	II.1	47y	F, 74y	c.24G > C	PT		Severe
				c.516C > A	MM		
Family III	III.1	34y	M, 50y	c.542delCA	TM	Loss of hydrophobic domain	Severe
				c.542delCA	TM		
	III.2	35y	M, 45y	c.542delCA	TM		Severe
				c.542delCA	TM		
Family IV	IV.1	58y	F, 74y	c.497A > G	MM	Totally loss of lipase function	Mild
				c.1442C > T	MM	Partially loss of lipase function	
Family V	V.1	52y	F, 52y	c.659delT	D	Loss of hydrophobic domain and localization	Severe
				c.659delT	D		
Family VI	VI.1	1y	M, 26y	c.41-47del	D	Probably no protein production	Moderate
				c.41-47del	D		
Family VII	VII.1	40y	F, 53y	c.553-565del	D	Loss of hydrophobic domain	Moderate
				c.696 + 4 > G	SSM	Loss of lipase function	
Family VIII	VIII.1	40y	M, 60y	c.177 T > G	MM	Partially loss of lipase function	Moderate
				c.577A > T	MM	Partially loss of lipase function	
	VIII.2	35y	M, 50y	c.177 T > G	MM		Mild
				c.577A > T	MM		
	VIII.3	58y	F, 58y	c.177 T > G	MM		Mild
				c.577A > T	MM		
Family IX	IX.1	64y	M, 79y	c.570A > C	MM	Affect central domain	Mild
				c.570A > C	MM		
Family X	X.1	45y	M, 47y	c.714C > A	MM	Unknown	Mild
				c.714C > A	MM		
Family XI	XI.1	5y	M, 14y	c.865C > T	MM	Partially loss of lipase function	Asymptomatic
				c.424A > T	PT	Loss of hydrophobic domain	

LEGEND: *PT* protein truncation, *MM* Missense mutation, *TM* truncated mutation, *D* Deletion, *DT* transcription defect, *SSM* splice site mutation. Severe: loss of ambulation, use of wheelchair; Moderate: interference with daily activity; Mild: symptomatic but not interference with daily activity; Asymptomatic: hyperCKemia without symptoms

The mean delay from the onset of clinical manifestations to diagnosis was 16.75 years (range 3–32 years) in patients with NLSD-M and 28 years (range: 1–65 years) in patients with NLSD-I.

The prognosis was unfavorable in 2 NLSD-I patients, who died of liver failure at the ages of 69 and 45 years after unsuccessful liver transplant.

### Genetic data

All the Italian families harboring different gene mutations are summarized in Tables 2 and 3, together with data on the severity of the clinical involvement for each patient. All homozygous patients were born from consanguineous parents. We identified 10 different mutations in 15 NLSD-M patients [5, 7–12], 5 of whom were homozygous and 10 heterozygous.

Mutations were found to be missense in 6 (55%) patients, nonsense in 3 (27%) and frameshift variants in 2 (18%). In one, previously described, case (pt.XV.1) no mutations were detected in either the PNPLA2 or CGI58 genes [6], but both ichthyosis and Jordans’ anomaly were present.

The molecular analysis of CGI58/ABHD5 revealed 4 different mutations in 5 subjects affected by NLSD-I [6, 13–16]. Two of these variations were nonsense (50%) while the other 2 were splice-site mutations (50%).

### Clinical data

The clinical data are summarized in Tables 1 and 2.

**Fig. 1:**
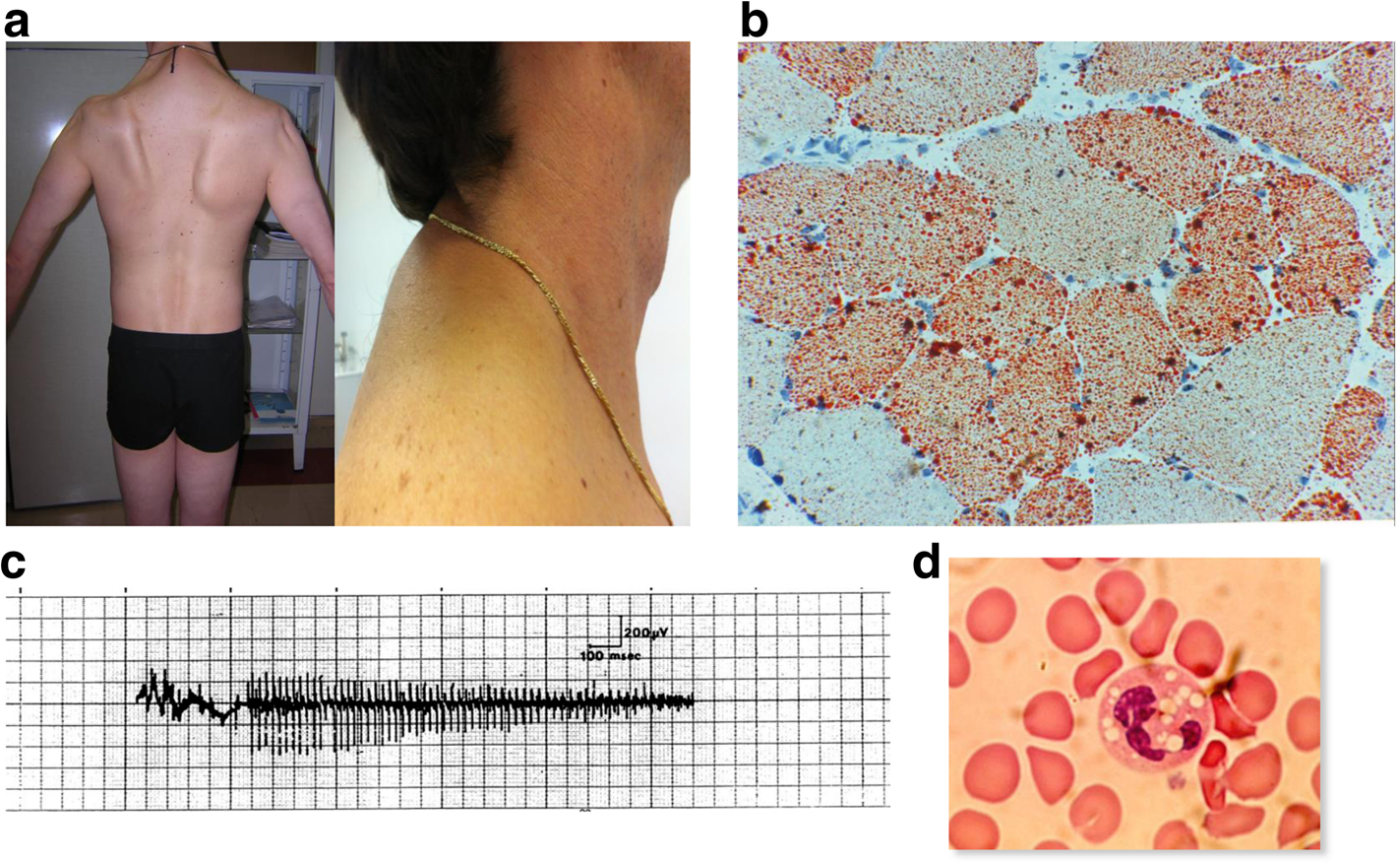
Legend: **a** Weakness and atrophy of proximal and axial muscles in pt. III.1. **b** Crysection of muscle O.R.O. stained with lipid increase in pt. I. **c** Myotonic discharge in patient with NLSD-M in pt. I.2. **d** Jordans’ anomaly in pt. I.1

All the patients with NLSD-M had prevalently myopathic symptoms consisting in weakness, which was accompanied by muscle atrophy in advanced cases. The onset was mainly asymmetric and in the upper limbs. The limbs were affected in all the cases, and the axial muscles, particularly the neck extensors, were also frequently weak and atrophic (Fig. 1a). In the early stages of disease, the proximal arm and leg muscles were often involved, while the distal muscles were always clinically involved in the advanced stages. Muscle weakness represented the first diagnostic symptom in all the NLSD-M patients: after a median disease duration of 30.6 years (15–50 year), 5 of the 21 patients lost their ability to walk autonomously (pts. I.1, II.1, III.2, V.1 with NLSD-M and pt. XIII.1 with NLSD-I) and now use assistive devices (4 are wheelchair-bound and pt. III.2 uses a walker), while 1 NLSD-M patient displayed difficulties in climbing stairs (pt. III.1). Fatigue was a constant symptom in all the patients with NLSD-M and in 3 patients with NLSD-I. Myalgia or cramps were present in 50% of the NLSD-M patients. Muscle atrophy was present in 8 NLSD-M and 3 NLSD-I patients. None of the 21 patients presented respiratory muscle involvement at the spirometry or at six-minute walk test (in subjects able to walk), nor ocular muscle involvement and/or difficulty in chewing and swallowing. The first sign in patients with NLSD-I was either liver disease or ichthyosis. The few asymptomatic patients had hyperCKemia. Ichthyosis was present in all patients with NLSD-I and transiently in only one patient with NLSD-M. None of the patients with NLSDs was obese; indeed, the majority were slender and only two had a slightly high BMI. Only one NLSD-I patient was short in stature.

### Serum test

Serum creatine kinase (CK) was high in all the NLSD-M cases and in 2/6 NLSD-I cases (the test was not performed in another 2 patients and was normal in the remaining 2), with CK levels ranging from 300 to 5700 U/l (average 1000). Routine blood tests showed normal cholesterol levels in all but 2 NLSD-M patients. Triglycerides were normal in all the patients but 1 with NLSD-I and 2 with NLSD-M. Mild hyperglycaemia and/or glycosuria were found in 4/15 NLSD-M and 1/6 NLDS-I patients. Jordans’ anomaly, which was tested in all the patients, was found in 100% of both NLSD-M and NLSD-I patients (Fig. 1b), and even in one case in which the genetic tests for PNPLA2 and CGI58/ABHD5 failed to detect mutations; we had hypothesized the involvement of another undefined gene in the triglyceride pathway in this last case (pt.XV.1). The percentage of leukocytes with lipid droplets varied from 10% (pt. IV.1) to 100% (Table 1), and correlated with disease severity though not with the patients’ age or disease duration.

### Biopsies

Muscle tissue histology in both NLSD-M and NLSD-I patients revealed mild atrophy and vacuolization of fibers, though without any increase in connective or adipose tissue (Fig. 1b).

No cellular infiltrates or significant necrosis were detected. Lipid droplets in the cytoplasm of muscle fibers were detected by means of optic microscopy in 93% of the muscle biopsies and were positive for O.R.O. staining. One case with NLSD-M (pt. IV.1 in Table 1) did not display any significant increase in lipid droplets in muscle fibers. Two patients also displayed a few ragged red fibers. A skin biopsy performed in 6 NLSD-M and 2 NLSD-I patients, stained with O.R.O., revealed excessive lipid droplet storage in all cases.

### Electromyographic studies

EMG revealed neurogenic alterations (increased MUP amplitude, spontaneous activity) in 3/15 NLSD-M patients, myopathic alterations (motor unit potentials of reduced amplitude and short duration) in 3/15 NLSD-M patients, myotonic discharges in 5/15 NLSD-M and 1/6 NLSD-I patients, and mixed pictures (neurogenic/myopathic) in 2/15 NLSD-M and 1/6 NLSD-I patients. Sensory and motor nerve conduction were normal in all the patients. Electrophysiological studies were not performed in 4/6 patients with NLSD-I because the patients were either too young or asymptomatic. The EMG revealed myotonic discharges in pt. IV.1 [9], in whom the muscle biopsy was been instead found normal (Fig. 1d) [14].

### Cardiological evaluation

The heart was examined in all the patients in both groups by means of an echocardiography and ECG; Holter ECG was also performed in 11 of the 21 patients (I.1, I.2, II.1, III.1, III.2, IV.1, V.1, VI.1, X.1, XII.1, XII.2) (Tables 1 and 2). Echocardiography documented cardiomyopathy with lipid infiltration in 6/15 patients with NLSD-M and 1/6 with NLSD-I, and was normal in 9/15 patients with NLSD-M. The most common echocardiographic alteration was ventricular hypertrophy. In one tested patient (III.1) cardiac MRI showed lipid infiltration; this is the only one patient carrying a defibrillator for severe arrhythmia.

### Eye and audiometric evaluation

A juvenile cataract was observed in 1/6 NLSD-I patients (XIV.1) and in one adult NLSD-M patient (IV.1). Deafness was present in only 2 adult NLSD-M patients (IX.1, XIII.1).

### CNS and psychiatric signs

All the patients had attended school and the majority were able to work. Ten NLSD-M patients (I.1, I.2, III.1, III.2, IV.1, V.1, VII.1, VIII.1, IX.1, X.1) also underwent a brain MRI, which revealed mild, non-specific, gliotic changes. Three patients with NLSD-M had psychiatric disturbances consisting of anxiety or paranoid personality (pts. III.1, IV.1, V.1).

One NLSD-M patient had intellectual disability and behavioral problems (pt. XIII.1). Intellectual disability was not present in any of our NLSD-I patients.

## Discussion

Previous studies on NLSDs have been conducted either on single cases or on very small cohorts of patients [17–22]. This multicentre study is the most comprehensive study on a specific population and the longest descriptive study on the natural history of patients with NLSDs. Although our data are likely to reliably represent the incidence of NLSD cases in Italy, it is impossible to determine the prevalence of the disease based on these data because very mild cases as well as a poor knowledge of this disease may lead to the disease being underdiagnosed. The difficulties encountered in making a diagnosis are due to the heterogeneous clinical presentation of NLSDs. The delayed diagnosis is due both to the lack of knowledge of the disease, even among experts in neuromuscular disorders, and to the difficulties in conducting a genetic study. In our experience, Jordans’ anomaly represents an inexpensive, reliable, practical biomarker of the disease in both, NLSD type M and I, as it was found to be present, to varying extents, in 100% of the patients tested.

All the Italian families enrolled in this study harbored a private mutation, which points to a high probability of gene mutations and polymorphisms with varying enzymatic functional properties [23]. The variability of the clinical phenotype suggests that the functional study of the mutations involved in this disease should be encouraged to collect information on the activity of the protein as it may be useful for prognostic purposes [5, 8, 9]. Our findings show that the spectrum of clinical severity is wider than previously reported. We identified subjects with a very late presentation in advanced age as well as subjects with a severe phenotype under the age of 40 years. We observed that patients diagnosed with NLSD-M may pass from normal activity to loss of self-sufficiency within a few years, as demonstrated by three patients (I.1, III.2 and V.1) in whom the disability progressed very rapidly, from disease onset to loss of ambulation and inability to handle objects, over a ten-year period. Several of our NLSD-M patients also displayed cardiac involvement, which did not however generally require therapies other than antihypertensive treatment, and only in one case a defibrillator for cardiac arrhythmia. This study suggests that the Italian phenotype is different from that observed in subjects from the Far East [24], in whom cardiac involvement seems to be the main clinical feature and often leads to heart transplantation. We observed that cardiac involvement in our patient series is independent of age and disease duration. The presence of associated features, such as intellectual disability and deafness, was not significant in our population.

In our cohort, the prognosis for patients with NLSD type I appeared to be more severe than that for patients with NLSD type M. Although life expectancy in our series of Italian patients with NLSD-M was normal, there was a significant reduction in the quality of life owing to motor disabilities in this group, with approximately one fourth of the cases suffering a loss of ambulation and one patient requiring an electric device. NLSD-I patients instead had a worse prognosis in terms of life expectancy, with two deaths due to hepatic failure caused by lipid infiltration. Although alterations in TG metabolism caused by ATGL/CGI58 mutations are known to lead to oxidative metabolism abnormalities [25], the pathogenesis of muscle damage in NLSDs has not yet been fully understood. In effect, muscle atrophy, present even in the early stages of disease, cannot be ascribed exclusively to metabolic defects. Unlike leukocytes, in which the proportion of lipids correlates with the severity of the disease, muscle lipid accumulation does not always correlate with the motor impairment. Indeed, as reported previously by other authors [12, 26, 27], we had patients (XI, XII.1, XII.2), with high lipid storage in muscle who were asymptomatic. Only one of our patients (IV.1), who did not have a significant accumulation of lipids in muscle, displayed EMG abnormalities, mild hyperCKemia, weakness and fatigability in the absence of atrophy.

The correlation between the phenotype and genotype in NLSDs cannot be easily investigated.

The number of patients with NLSD-I was too small for conclusive results about phenotype/genotype correlation (Table 2). A recent Turkish report on this question failed to detect any meaningful correlations [28]. In NLSD-M, an evaluation of residual enzymatic activity in vitro can predict the type of mutation and provides general information on the severity of the disease. Nevertheless, we observed that there was a marked variability in disease expression even within the same family (see families III and VIII), which contained younger members who were unexpectedly affected largely than their older siblings.

## Conclusions

The severity of the clinical involvement in NLSD-M seems to depend partially on the type of mutation and on residual enzymatic activity, as reported in a previous study [20]. The same mutation in our cohort was found to result in different phenotypes; it is conceivable that epigenetic factors, such as the environment and lifestyle, which are known to affect muscle activity, and diet (e.g. containing varying amounts of different types of lipids), may also play an important role. We cannot rule out the possibility that other genes involved in the complex system of lipid metabolism also affect disease expression in NLSD-M. This study provides valuable information on the prognosis in this group of diseases, which may be used to counsel patients and improve the management and standards of care.

Lastly, we provide data on the progression of such diseases, which might be crucial for planning future clinical and therapeutic trials.

## Abbreviations

6MWT: Six Minutes Walking Test
CGI58: Abhydrolase domain containing 5 - ABHD5 (CGI-58)
CK: Creatine kinase
ECG: Electrocardiography
EMG: Electromyography
MAD: Medium chain acyl-CoA dehydrogenase deficiency
NLSD-I: Neutral Lipid Storage Disease type with Ichthyosis
NLSD-M: Neutral Lipid Storage Disease type with Myopathy
NLSDs: Neutral Lipid Storage Diseases
O.R.O.: Oil Red O
PNPLA2: Patatin-like phospholipase domain containing 2
TG: Triglycerides
